# Molybdenum Oxynitride Atomic Nanoclusters Bonded in Nanosheets of N-Doped Carbon Hierarchical Microspheres for Efficient Sodium Storage

**DOI:** 10.1007/s40820-022-00893-7

**Published:** 2022-08-13

**Authors:** Xiaona Pan, Baojuan Xi, Huibing Lu, Zhengchunyu Zhang, Xuguang An, Jie Liu, Jinkui Feng, Shenglin Xiong

**Affiliations:** 1 School of Chemistry and Chemical Engineering, State Key Laboratory of Crystal Materials, Shandong University, Jinan, 250100 People’s Republic of China; 2 School of Mechanical Engineering, Chengdu University, Chengdu, 610106 People’s Republic of China; 3 The State Key Laboratory of High Performance Ceramics and Superfine Microstructure, Shanghai Institute of Ceramics, Chinese Academy of Sciences, Shanghai, 200050 People’s Republic of China; 4 School of Materials Science and Engineering, Shandong University, Jinan, 250100 People’s Republic of China

**Keywords:** Molybdenum oxynitride, Atomic nanocluster, Hollow microspheres, Sodium-ion batteries

## Abstract

Transition metal nitrides have attracted considerable attention as great potential anode materials due to their excellent metallic conductivity and high theoretical specific capacity. However, their cycling performance is impeded by their instability caused by the reaction mechanism. Herein, we report the engineering and synthesis of a novel hybrid architecture composed of MoO_2.0_N_0.5_ atomic nanoclusters bonded in nanosheets of N-doped carbon hierarchical hollow microspheres (MoO_2.0_N_0.5_/NC) as an anode material for sodium-ion batteries. The facile self-templating strategy for the synthesis of MoO_2.0_N_0.5_/NC involves chemical polymerization and subsequent one-step calcination treatments. The design is beneficial to improve the electrochemical kinetics, buffer the volume variation of electrodes during cycling, and provide more interfacial active sites for sodium uptake. Due to these unique structural and compositional merits, these MoO_2.0_N_0.5_/NC exhibits excellent sodium storage performance in terms of superior rate capability and stable long cycle life. The work shows a feasible and effective way to design novel host candidates and solve the long-term cycling stability issues for sodium-ion batteries.

**Supplementary Information:**

The online version contains supplementary material available at 10.1007/s40820-022-00893-7.

## Highlights

- MoO_2.0_N_0.5_ atomic nanoclusters bonded on nanosheets of N/C hierarchical hollow microsphere are synthesized via a facile self- templating strategy.
- Introduction of MoO_2.0_N_0.5_ atomic nanoclusters benefits the enhancement of electrochemical kinetic.
- Composite of MoO_2.0_N_0.5_/NC shows excellent electrochemical performance as an anode material for sodium-ion batteries.

**Supplementary Information:**

The online version contains supplementary material available at 10.1007/s40820-022-00893-7.

## Graphical abstract

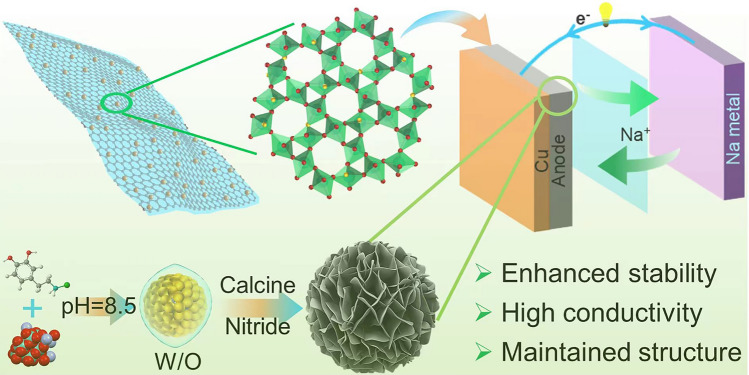

## Introduction

In recent years, sodium-ion batteries (SIBs) have undergone rapid development due to ultra-low-cost and abundant sodium sources, similar physicochemical properties as lithium metal, and low reduction potential, which could provide a competitive alternative to lithium storage systems [1, 2]. The recognition will become increasingly significant because of the diminution of economically attractive lithium sources [3, 4]. Taking inspiration from lithium storage, the scientific community has developed various anode material in past decades [5–8]. Graphite is the most promising anode materials for lithium energy storage [9, 10]. However, Na-ion requires enormous C-atoms to form a stable intercalation compound (e.g., NaC48, NaC64, NaC80) during intercalating into the graphene anode, resulting in an unpractical low reversible capacity [11]. Compared with graphite anodes, metal compounds exhibit intriguing reversible capacity because these compounds could store Na-ion by alloying and conversion reactions [12]. Moreover, these materials would consume fewer electrolytes than the carbon-based anodes due to their high mass density.

Recently, the molybdenum (Mo) compounds have been widely investigated because that among all these metal compounds, Mo-based compounds exhibit favorable crystal structure, considerable capacity low toxicity, and easily fabricated, especially good electrical conductivity [13, 14]. However, bare Mo-based compounds usually deliver inferior rate performance and short lifespan due to their insufficient electrical conductivity and huge volume expansion during the charging/discharging process. To solve these drawbacks, many modified methods have been developed, including constructing pore structures and hollow structures, which can promote the ion diffusion kinetic and suppress the volume expansion [15, 16]. Meanwhile, carbon-modified Mo-compounds could provide fast electron transport pathway, and the heteroatoms doping can increase their intrinsic conductivity and extremely facilitate the kinetics of Mo-based electrodes [17, 18]. Intriguingly, Mo-based nitrides have aroused considerable attention because of their high theoretical specific capacity and excellent electrical conductivity [19, 20]. Till now, however, the most synthesized Mo-based nitrides are in form of thin films, nanoparticles, nanowires, and nanobelts, which are different from metal oxides and metal sulfides with abundant nanostructures morphology. Although these 0- to 2-dimensional (0–2D) types of Mo-based nitrides display enhanced electrochemical properties, these materials still deliver unsatisfactory specific capacity and electrochemical properties owing to their inferior specific surface area and long transport paths of sodium ion during applying as SIBs anode [19, 21]. Consequently, the introduction of three-dimension (3D) porous nanostructure into Mo-based nitrides could be expected to further enhance their electrochemical properties and electrical conductivity.

A crucial strategy to solve inherent issues of SIBs and achieve better performance is to utilize nanostructured anode materials. Compared with traditional nanomaterials, atomic nanoclusters show excellent strongly active, huge surface expose, and low surficial atoms coordinate and have been remarkably used in energy storage and conversion system [22, 23]. To date, however, the nanocluster-based anode materials, which are atom-discernable but not a group of nanoparticle assemblages, have yet to study for the electrode preparation for SIBs. Herein, we designed and synthesized a composite of MoO_2.0_N_0.5_ atomic nanoclusters and N-doped carbon hierarchical hollow microspheres (MoO_2.0_N_0.5_/NC), which was featured by atom-defined MoO_2.0_N_0.5_ atomic nanoclusters anchored on the surface of N-doped carbon nanosheets. As-prepared MoO_2.0_N_0.5_/NC hollow microspheres as anode displayed some preponderances. First, MoO_2.0_N_0.5_ atomic nanoclusters played an important role in enhancement of electrochemical kinetic. Second, the constructed N-doped carbon hollow microspheres were exposed to more surface-active sites to mobilize the ions and shorten the Na-ion diffusion distance. Meanwhile, the inherent excellent electrical conductivity and improved rapid electron transfer jointly facilitated the reaction kinetics of desodiation/sodiation. As a result, the MoO_2.0_N_0.5_/NC composite anode displayed significantly enhanced electrochemical performance with high capacity, excellent rate performance, long-term cycling stability, and a great opportunity for high-performance SIBs.

## Experimental

### Sample Preparation

#### Synthesis of Mo-Polydopamine Hybrid

2 mmol ammonium molybdate tetrahydrate ((NH_4_)_6_Mo_7_O_24_·4H_2_O, AMT) and 1.6 mmol dopamine hydrochloride (DMH) were dissolved in 80 mL deionized water and stirred. After 30 min continues stirring, 150 mL ethanol was added into the mixture, and the pH was held constantly at 8.5 ± 0.1 by ammonium hydroxide to turn the base solution frequently and stirred for 2 h. The dark red precipitation was collected by centrifugation, washed with ethanol for three times, and finally filtrated and dried in a vacuum oven at 60 °C for 24 h.

#### Fabrication of MoO_2.5_/NC Microsphere

Mo-polydopamine hybrid was placed in a tube furnace with an Al_2_O_3_-boat. The tube furnace was heated at 600 °C for 3 h with a heating rate of 5 °C min^−1^ in Ar flow, and then naturally cooled down to 20 °C.

#### Fabrication of MoO_2.0_N_0.5_/NC Microsphere

The obtained MoO_2.5_/NC and C_3_N_4_ were mixed into ethanol with a weight ratio of 1/5, and grinded with a glass rod. Then, the mixture was dried at 60 °C for 6 h. The dried mixture was placed in a tube furnace and heated at 600 °C for 3 h in Ar atmosphere with a heating rate of 5 °C min^−1^.

### Materials Characterization

The morphologies and structures of the prepared materials were performed on a Gemini 300 field emission scanning electron microscope (SEM) with an accelerated voltage of 5 kV and a field emission transmission electron microscope (TEM, JEOL JEM-ARM 200F, FEI TalosF200x) operated at 200 kV with a field-emission scanning electron microscope (EDS, super-X EDS) at 5 kV. The crystalline structures of the products were conducted by X-ray diffraction (XRD) with a wavelength of 1.5418 Å. The nitrogen adsorption-desorption isotherm was carried out to obtain the texture properties of the products at 77 K on a Micromeritics analyzer (ASAP-2000 HD 88). Thermogravimetric analysis (TGA) was applied to test the carbon content of the products by heating specimen from room temperature to 650 °C with a rate of 10 °C min^−1^ under the air flow on a TA instrument (Mettler Toledo TGA/SDTA851). Raman spectra of the products were performed on a confocal laser micro-Raman spectrometer (JY LABRAM-HR) with a wavelength of 514.5 nm. FT-IR spectra were measured on a Fourier transform infrared spectrometer (FT-IR, Bruker, Tensor II) in a wavenumber range of 400–4000 cm^−1^. The composition of the products was detected on an X-ray photoelectron spectroscopy (XPS, Thermo ESCALAB 250Xi). The C 1* s* peak at 284.8 eV is the reference.

### Cell Performance Testing

The MoO_2.5_/NC and MoO_2.0_N_0.5_/NC electrodes were prepared by mixing a slurry of MoO_2.5_/NC (or MoO_2.0_N_0.5_/NC), carboxy methyl cellulose (CMC), and carbon black with a mass ratio of 8/1/1 in the N-methyl-2-pyrrolidone (NMP) solvent. The slurry was cast onto a Cu foil (purity: 99.95%, Alfa Aesar, China) with a thickness of 15 µm. Then, the anode electrodes were transferred into a vacuum oven to dry at 80 °C for overnight. The anode electrodes were punched into 12-mm-diameter round disk and transferred into an Ar-filled glovebox (O_2_ < 0.1 ppm, H_2_O < 0.1 ppm). The mass loading of active materials of MoO_2.5_/NC (or MoO_2.0_N_0.5_/NC) was around 1.5 g cm^−2^. The coin cells were assembled in a glovebox with Ar-filled, where sodium foil was counter and reference electrode, glass fiber was separator, and 1 M NaClO_4_/5 vol% fluoroethylene carbonate (FEC)/ethylene carbonate (EC) -diethylene carbonate (DEC) (v/v = 1/1) as electrolytes. The sodium-ion batteries were galvanostatic charged/discharged on a Land-CT2001A in the range of 0.1–3 V (*vs*. Na^+^/Na) with a various current density of 0.1–20 A g^−1^. All coin cells were activated for three cycles at 0.1 A g^−1^ before high current density. Potentiostat electrochemical impedance spectroscopy (EIS) was used to investigate the resistance of coin cells with a frequency range between 0.01 Hz and 100 kHz and an AC amplitude of 5 mV. The EIS was performed on the sodium-ion batteries before and after various cycles with a current density of 1 A g^−1^. Cyclic voltammetry (CV) was measured on a CHI760E electrochemical workstation in a voltage range of 0.1–3 V (*vs*. Na^+^/Na).

## Results and Discussion

### Physical Characterization

**Fig. 1:**
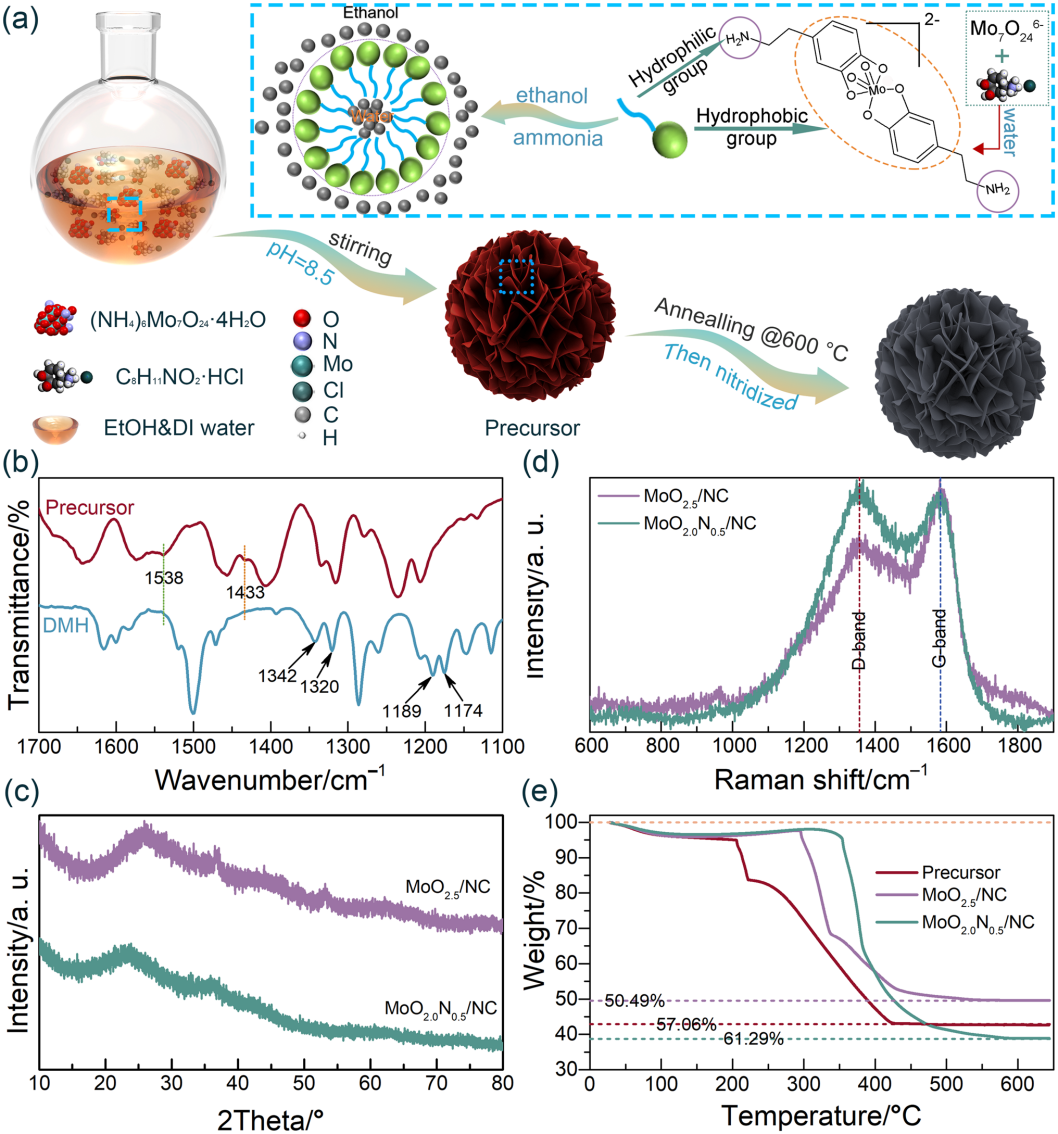
**a** Scheme of synthesis of MoO_2.0_N_0.5_/NC hollow microspheres. **b** FT-IR spectra for MoO_2.5_/NC and MoO_2.0_N_0.5_/NC. **c** XRD patterns and **d** Raman spectra for MoO_2.5_/NC and MoO_2.0_N_0.5_/NC. **e** TGA curves for precursor (Mo-polydopamine hybrid), MoO_2.5_/NC, and MoO_2.0_N_0.5_/NC

The composite of MoO_2.0_N_0.5_/NC hollow microsphere is synthesized by a simple polymerization at room temperature combined with calcination, wherein MoO_2.0_N_0.5_ atomic nanoclusters are uniformly distributed on the nanosheets of the N-doped carbon hierarchical hollow microsphere. The schematic diagram of the fabrication process is illustrated in Fig. 1a. As shown in Fig. 1a, we mix ammonium molybdate and dopamine hydrochloride in an aqueous solution with a molar ratio of 8:1, which shows a clarified solution with orange–red color. The dopamine HCl is chelated with molybdate-ion. Mo-dopamine chelate has very low solubility in ethanol. Thus, with the addition of ethanol, H_2_O/ethanol interface is gradually formed, where the hydrophobic groups of Mo-dopamine chelate toward ethanol and the hydrophilic groups orient inward water, and the solution turns to orange–yellow suspension. With ammonia added into the solution to adjust the pH, the self-polymerization of the dopamine is initiated along the water/ethanol interface.

Mo-polydopamine hybrid (precursor) is synthesized by mixing AMT and DMH. Polydopamine as a carbon source is prepared under alkaline conditions via adding ammonium hydroxide. The molecular of dopamine hydrochloride has plenty of amino and catechol groups, which can form chelate components with transition metal ions and metal oxyanions by the polymerized reaction [24]. FT-IR spectra of DMH and precursor are shown in Fig. 1b (full spectra in Fig. S1). It is observed that DMH has an absorption band at 1342 and 1320 cm^−1^, which can be attributed to the CH_2_ and C-O–H bending vibration, while the absorption band at 1189 and 1174 cm^−1^, which can be assigned to the C-O and C–C stretching vibration, separately [25]. For the Mo-polydopamine hybrid, there are plenty of catechol and amino groups that can chelate transition-metal ion and metal-containing oxyanion by self-polymerization can be observed in FT-IR spectra. Moreover, the new absorption bands at 1538 and 1433 cm^−1^ emerge, which indicates that an indole structure is formed [25, 26]. To obtain MoO_2.0_N_0.5_/NC, Mo-polydopamine hybrid is calcined at elevated temperature on a tube furnace under an Ar flow. For comparison, MoO_2.5_/NC was also synthesized in the same condition without addition of C_3_N_4_. The synthesis detail is explained in the experimental section.

The crystallinity of prepared MoO_2.5_/NC and MoO_2.0_N_0.5_/NC is demonstrated by the XRD. As shown in Fig. 1c, both MoO_2.5_/NC and MoO_2.0_N_0.5_/NC display a broaden peak at 25.3°, which is attributed to carbon. It can be observed in Fig. 1c that MoO_2.5_/NC is a crystalline material and there is amount of carbon content resulting in a series of weak crystalline peaks*.* While mixed MoO_2.5_/NC with C_3_N_4_ to introduce N atoms into MoO_2.5_/NC, the crystal phase of product MoO_2.0_N_0.5_/NC is changed to an amorphous material [27]. Figure 1d represents the Raman spectra of MoO_2.5_/NC and MoO_2.0_N_0.5_/NC to analyze the defects and graphitization. There are two strong peaks at ~ 1356 and ~ 1584 cm^−1^, corresponding to D-bond and G-bond of carbon. Generally, the degree of defects of carbon materials is verified by the intensity ratio of *I*_D_/*I*_G_ [28, 29]. Here, a higher value of *I*_D_/*I*_G_ of MoO_2.0_N_0.5_/NC represents that the structure has more active defect sites for intercalation/extraction of Na^+^ caused by the introduction of MoO_2.0_N_0.5_ atomic nanoclusters and heteroatoms [30].

TGA is carried out to determine the MoO_2.5_ and MoO_2.5_N_0.5_ content in the composite (Fig. 1e). The weight loss before 350 °C can be attributed to oxidation of MoO_2.5_ and MoO_2.0_N_0.5_ to MoO_3_ and NO_2_. The carbon content of MoO_2.5_/NC and MoO_2.0_N_0.5_/NC in the composites is obtained as 53.3% and 62.9%, respectively (the calculation details are shown in Fig. S2). The overall amount of C, N, O, and Mo of MoO_2.5_/NC and MoO_2.0_N_0.5_/NC is listed in Table S1, which is obtained from the CHN elemental analysis and inductively coupled plasma mass spectrometer (ICP-MS) measurement. The overall amount of C in the MoO_2.5_/NC and MoO_2.0_N_0.5_/NC is estimated to be 50.7% and 54.4%. More importantly, the amount of N element of MoO_2.0_N_0.5_/NC is 2.36%, while that of MoO_2.5_/NC is only 0.33%, which is illustrated that N is successfully doped into MoO_2.5_/NC.

**Fig. 2:**
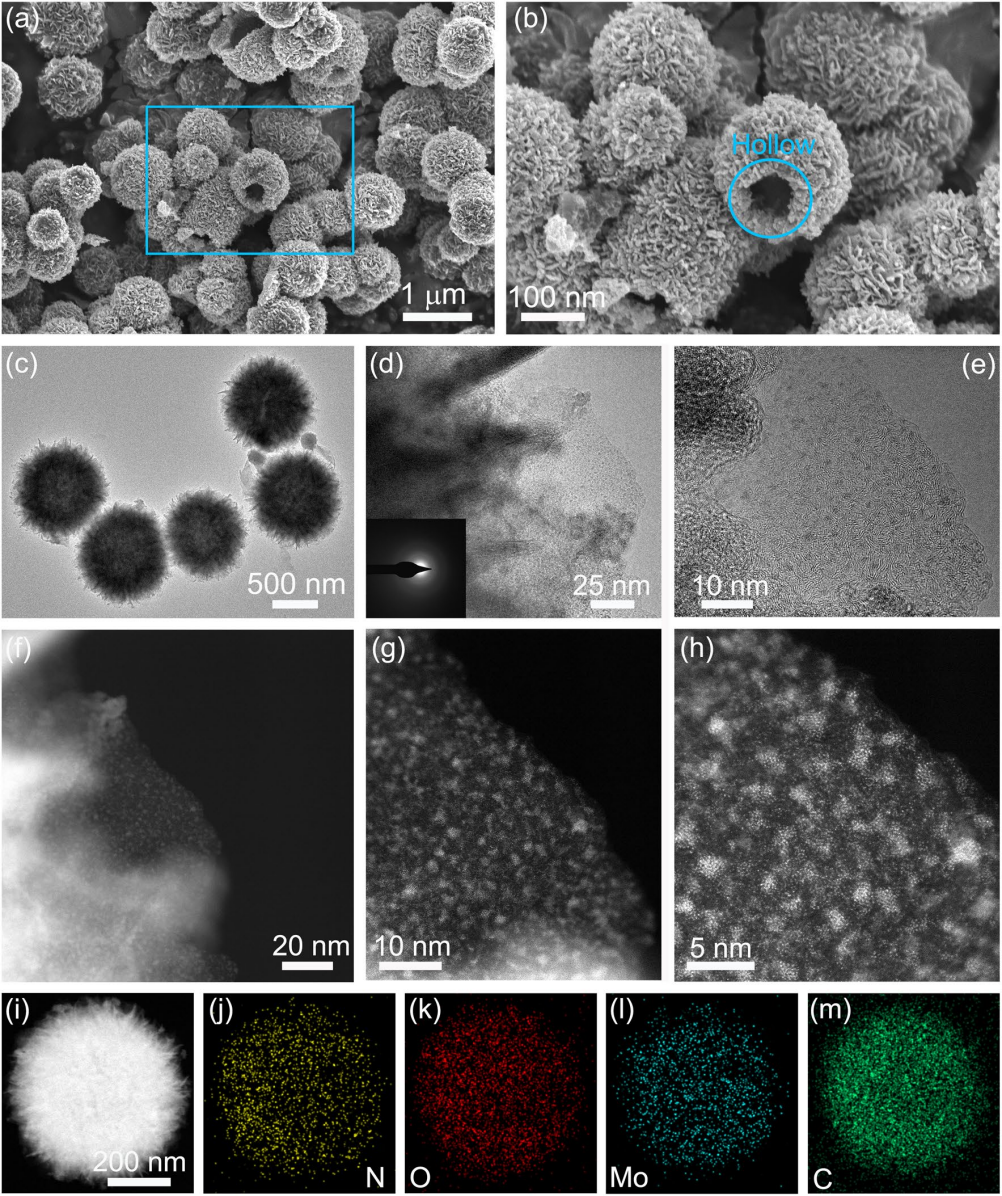
Morphological and structural characterization of MoO_2.0_N_0.5_/NC. **a, b** FESEM images. **c** TEM images. **d, e** HRTEM images. **f–h** HAADF-STEM images. **i** STEM image, and the corresponding EDX mappings of **j** N, **k** O, **l** Mo, and **m** C. The insert in **d** is the SAED pattern of MoO_2.0_N_0.5_/NC

To verify the porous structural characterization of MoO_2.5_/NC and MoO_2.0_N_0.5_/NC, the nitrogen adsorption/desorption measurement is performed and the results are shown in Fig. S3. The N_2_-sorption isotherms of MoO_2.5_/NC and MoO_2.0_N_0.5_/NC are typical type III isotherm. The type-H_3_ hysteresis loop appears at a relative pressure *P*/*P*_0_ over 4.5, indicating that slit-like pores are constructed by flake particles stack. By Brunauer–Emmett–Teller model, the specific surface area of MoO_2.0_N_0.5_/NC is calculated with a value of 63.4 m^2^ g^−1^, which is higher than that of MoO_2.5_/NC of 42.9 m^2^ g^−1^. It can be explained that the introduction of MoO_2.0_N_0.5_ atomic nanoclusters effectively facilities the stacking and agglomeration and results in the formation of porous microspheres. Additionally, Fig. S3d shows the pore size distribution curves according to the Barrett–Joyner–Halenda model. The pore size of both MoO_2.5_/NC and MoO_2.0_N_0.5_/NC mainly distributes in a range of 3–15 nm and an obvious increase of specific pore volume with N-doping (Fig. S3d). It reveals that MoO_2.0_N_0.5_/NC has more abundant mesopores than MoO_2.5_/NC. The total pore volume of MoO_2.0_N_0.5_/NC is 0.36 cm^3^ g^−1^, which is larger than that of MoO_2.5_/NC of 0.26 cm^3^ g^−1^. This can be explained that carbon layers of MoO_2.0_N_0.5_/NC provide more mesopores and active defect site, which agrees with results of Raman spectra. The MoO_2.0_N_0.5_/NC micro- and nanostructure has a large specific area and rich mesopores, which is beneficial to increase the contact area between the electrode and electrolytes and improve the penetration of electrolytes [31]. The morphology and detailed microstructure of the as-synthesized MoO_2.0_N_0.5_/NC are examined by field emission scanning electron microscope (FESEM) and TEM as shown in Fig. 2, while that of precursor and MoO_2.5_/NC is exhibited in Figs. S4 and S5. It is noticed that hierarchical hollow microspheres are synthesized with a size of ~ 1 µm (Fig. S4). Furthermore, the morphologies of the hollow microsphere are perfectly maintained after calcinating at elevated temperatures (Figs. 2a–b and S5). Figures S5 and 2a–b present the morphologies of MoO_2.5_/NC and MoO_2.0_N_0.5_/NC, indicating high yield hollow microsphere and the unchanged size. FESEM images of precursor and MoO_2.5_/NC and their corresponding energy-dispersive X-ray spectroscopy (EDX) spectrum elemental mapping exhibit that Mo, C, N, and O elements are a uniform spatial distribution throughout the entire hollow microsphere (Figs. S4–S5). As shown in Fig. 2a–c, it is obvious to observe that the MoO_2.0_N_0.5_/NC is a hollow microsphere and the 3D network of MoO_2.0_N_0.5_/NC has abundant voids between the nanosheets, which can enhance the contact between the active materials and electrolytes and alleviate the volume expansion. Importantly, the well-defined and ultrafine MoO_2.0_N_0.5_ nanoparticles are uniformly distributed on the N-doped carbon nanosheets (Fig. 2d). Figure 2e displays the high-resolution TEM image of MoO_2.0_N_0.5_/NC and reveals that MoO_2.0_N_0.5_ atom-discernable clusters with evenly uniform sizes without continuous crystal lattice stripes are homogeneously distributed on the N-doped carbon nanosheets, which reveals the lack of long-range atomic structure, *i.e.,* amorphous structure and is consistent with the results of XRD [32]. Furthermore, insert patterns in Fig. 2d of the selected area electron diffraction (SAED) show the weak ring, further indicating that these materials have an amorphous structure. The size of distributed MoO_2.0_N_0.5_ atomic nanoclusters is further estimated by the atomic-resolution high-angle annular dark-field scanning TEM (HAADF-STEM) images, *i.e.,* ~ 1 nm (Fig. 2f–g). Moreover, some discrete bright dots can be observed in Fig. 2h, indicating that trace single-atom MoO_2.0_N_0.5_ existed during the calcining process. The STEM image of MoO_2.0_N_0.5_/NC and corresponding EDX spectrum elemental mapping exhibit that Mo, C, N, and O elements are a homogenous distribution throughout the entire hollow microsphere (Fig. 2i–m). The EDX spectra obtained for an undoped and a N-doped single hollow microsphere show the stoichiometry of Mo/O of 1/2.5 and Mo/O/N of 1/2.0/0.5 (Fig. S6), respectively. Combined with the CHN tests and ICP-MS measurements, the composition is confirmed as MoO_2.5_/NC and MoO_2.0_N_0.5_/NC. The EDX spectrum from a single N-doped hollow microsphere exhibits a N-atomic percentage of 2.76% (Fig. S6b), which is increased compared to an undoped one.

**Fig. 3:**
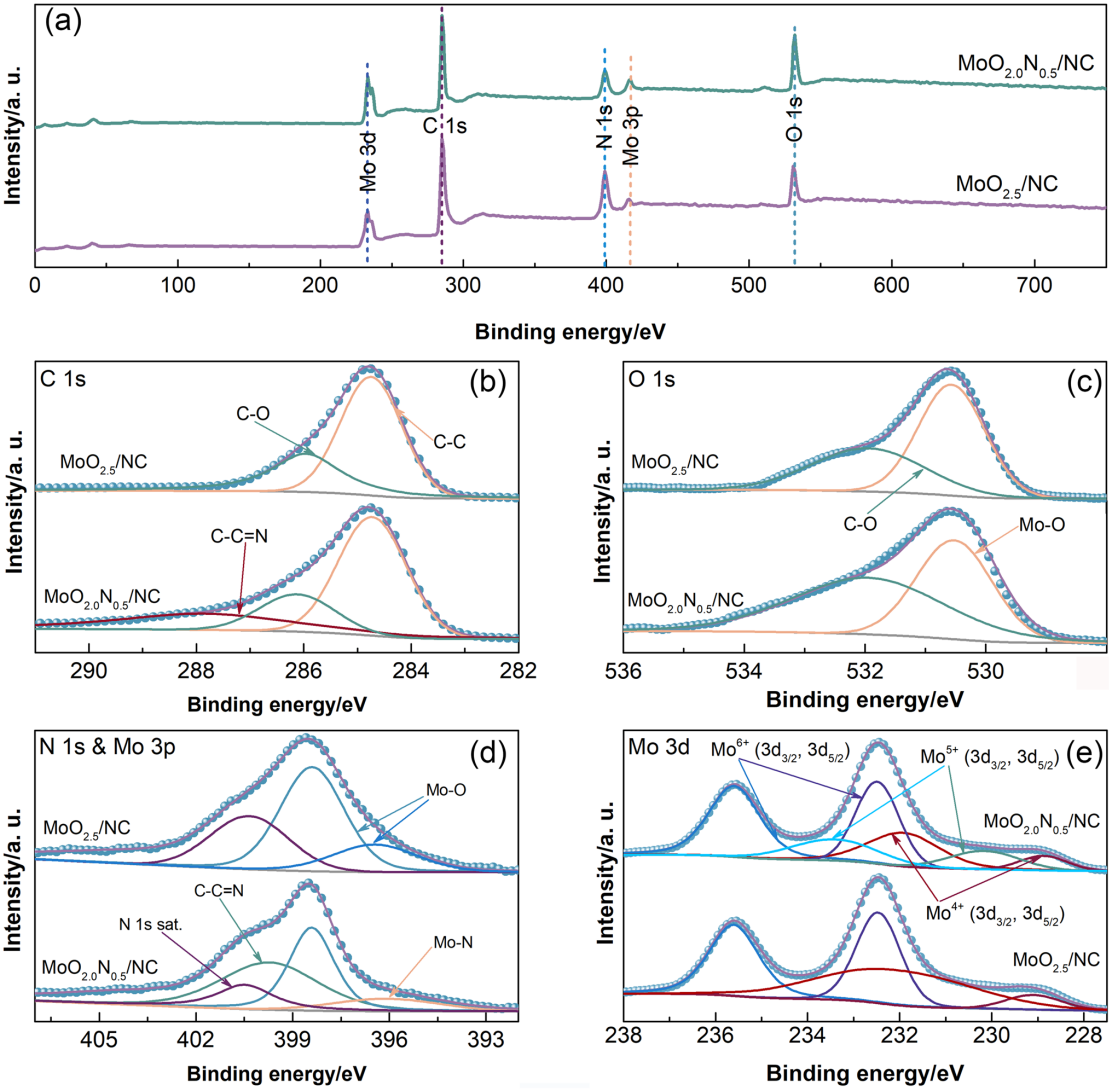
**a** XPS spectra of MoO_2.5_/NC and MoO_2.0_N_0.5_/NC; high-resolution XPS spectra of C **b** 1* s*, **c** O 1* s*, **d** N 1* s* & Mo 3*p*, and **e** Mo 3*d*

XPS is carried out to analyze the MoO_2.5_/NC and MoO_2.0_N_0.5_/NC compositions of elements and their chemical state. As shown in Fig. 3, the elements of C, O, N, and Mo are detected. The XPS survey spectra and high-resolution spectra of common metal in carbon indicate that MoO_2.5_/NC and MoO_2.0_N_0.5_/NC contain only C, O, N, and Mo without detectable impurity on the surface of the compositions (Fig. 3a). The standard peak of C 1* s* is at 284.8 eV as the reference. For C 1* s* and O 1* s* spectra, the peaks at 286.1 and ~ 532.5 eV are attributed to C-O bond. The new peaks at 288 and 399.8 eV of Mo_2.0_N_0.5_/NC hollow microsphere are assigned to the *sp*^2^-bond N in triazine rings (C-N = C) [33]. The fitting peak of O 1* s* binding energy is concerned at 530.5 eV, which is typical for Mo–O coordination, indicating the MoO_x_ (x = 2 ~ 3) components form on the surface [34]. For high-resolution XPS spectra of N 1* s* and Mo 3*p* (Fig. 3d), the distinct N and Mo could be deconvoluted into three peaks for MoO_2.5_/NC and MoO_2.0_N_0.5_/NC, illustrating the presence of diverse chemical states of N and Mo. The peak at binding energy of 400.4, 399.8, 398.4, 396.4, and 395.8 eV can be assigned to the N 1* s* satellite, C–C = N, Mo–O, Mo–O, and Mo–N bonds [35]. As shown in Fig. 3e, high-resolution XPS spectra of Mo 3d exhibits two characteristic peaks of Mo^6+^ 3d_3/2_ and Mo^6+^ 3d_5/2_ centered at 232.9 and 236.0 eV, respectively. The results correspond to the standard XPS spectrum for MoO_x_ (x = 2 ~ 3) (Mo^4+^ 3d_5/2_ at 232.4 eV, Mo^6+^ 3d_5/2_ at 236.0 eV) [36]. For MoO_2.0_N_0.5_/NC materials, the new peaks at 230.2 and 233.5 eV can also be observed, which are attributed to Mo^5+^ 3d_3/2_ and Mo^5+^ 3d_5/2_, separately [37]. On the surface of the hollow microsphere MoO_2.0_N_0.5_/NC, there is amount of Mo^5+^ appears after N-doped, indicating that the MoO_2.0_N_0.5_ atomic nanoclusters have been successfully introduced into nanosheets of NC hollow microspheres.

### Electrochemical Results

**Fig. 4:**
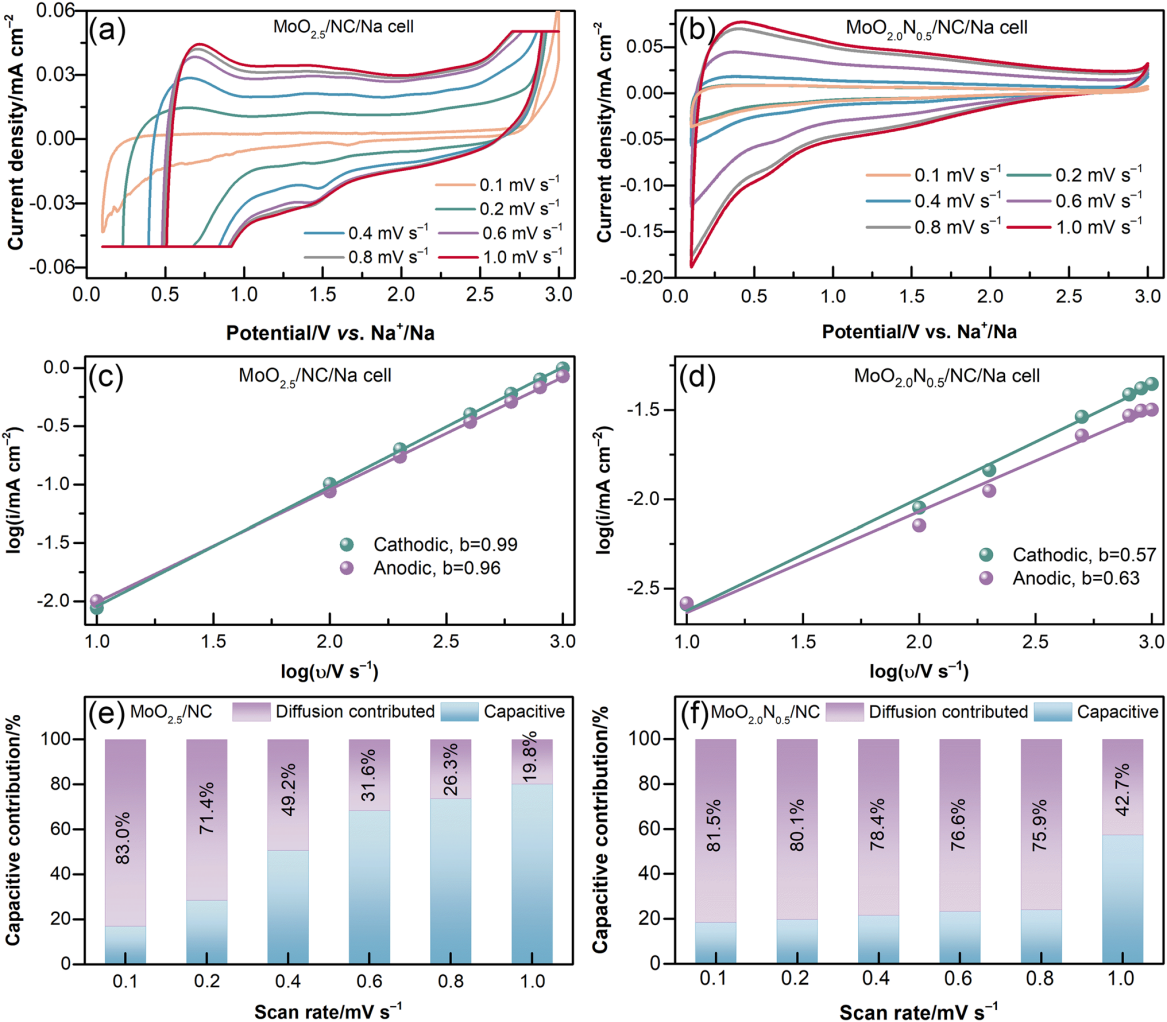
Kinetics analysis of the MoO_2.5_/NC and MoO_2.0_N_0.5_/NC electrode. CV curves for **a** MoO_2.5_/NC/Na cells and **b** MoO_2.0_N_0.5_/NC/Na cells with a various scan rate. *b*-value analysis using the relationship between the anodic and cathodic peak currents and scan rates: **c** MoO_2.5_/NC and **d** MoO_2.0_N_0.5_/NC. Contribution ratio of the capacitive and diffusion-controlled at various scan rate for **e** MoO_2.5_/NC and **f** MoO_2.0_N_0.5_/NC anodes

To demonstrate the effect of MoO_2.0_N_0.5_ atomic nanoclusters and heteroatoms in the anode materials for mobilizing ions, the kinetics behaviors of MoO_2.5_/NC and MoO_2.0_N_0.5_/NC are systematically explored for SIBs. Figure 4a-b displays the initial CV curves of the MoO_2.5_/NC and MoO_2.0_N_0.5_/NC as electrodes with various scan rates of 0.01–1 mV s^−1^ in a voltage range between 0.1 and 3.0 V (*vs.* Na^+^/Na). The reaction kinetics of MoO_2.0_N_0.5_/NC in SIBs are investigated by CV tests with various scan rates (υ). The power-law formula has been used to qualitatively analyze the capacitance effect. The peak current density (*i*) has a relationship with the scan rate *υ* as an equation of *i* = aυ^*b*^ [37, 38]. The value of *b* can be calculated by the function of log(*i*) and log(*υ*). The curves of log(*i*)–log(*υ*) of MoO_2.5_/NC and MoO_2.0_N_0.5_/NC electrodes are shown in Fig. 4c-d, respectively. The *b* values of the cathodic and anodic peaks are obtained and the values are between 0.5 and 1, illustrating a mixed mechanism [39]. Particularly, the cathodic and anodic *b* values of MoO_2.0_N_0.5_/NC are around 0.6, indicating the increase of the diffusion-controlled contribution with the introduction of heteroatoms, which probably results in enhanced ion diffusion. Moreover, the diffusion-controlled contribution (*k*_1_υ) and capacitive contribution (*k*_2_υ^1/2^) are the current response *i*_r_ at a fixed voltage *V* and estimated by the following equation: *i*_r_(*V*) = *k*_1_υ + *k*_2_υ^1/2^ [40]. By obtaining the value of *k*_1_ and *k*_2_, the *i*_r_(*V*) can be calculated and divided into two parts, *i.e.,* diffusion-controlled *k*_1_ and capacitor-controlled *k*_2_. The capacitive contribution ratio at various scan rates is summarized in Figs. 4e–f and S7 provides the capacitive contribution ratio at 0.1 mV s^−1^. It is observed that the distributive capacitance of MoO_2.0_N_0.5_/NC is lower than that of MoO_2.5_/NC, which is further revealed that MoO_2.0_N_0.5_/NC electrodes have improved Na-ion diffusion.

The electrochemical characterization of MoO_2.0_N_0.5_/NC electrode is investigated by CV with a scan rate of 0.1 mV s^−1^ in a voltage range of 0.1–3 V (*vs.* Na^+^/Na) for the initial three cycles in Fig. S8. The CV curves show the initial prominent peaks centered at ~ 0.7 and 0.4 V, which is related to sodium-ion intercalation into MoO_2.0_N_0.5_ to form Na_x_MoO_2.0_N_0.5_ phase and the formation of solid electrolytes interphase (SEI) layers when MoO_2.0_N_0.5_/NC is employed as an anode for SIBs (shown in Fig. S8) [41–45]. The initial irreversible capacity of MoO_2.0_N_0.5_/NC electrode results from the formation of SEI layer, the decomposition of the electrolytes, and the irreversible sodium-ion insertion. As shown in Fig. S8, a cathodic peak at ~ 0.1 V and an anodic peak at 0.2 V can be ascribed to the sodium-ion intercalation and de-intercalation, respectively [46–48]. Furthermore, the initial anodic scan shows a several peaks between 1.2 ~ 1.8 V, which is caused by the multistep de-intercalation of Na_x_MoO_2.0_N_0.5_ to MoO_2.0_N_0.5_. After the initial cycle, the nearly overlapped CV curves of MoO_2.0_N_0.5_/NC electrode in SIBs demonstrate the high cycling reversibility, which can be attributed to N-doped.

Furthermore, Fig. S9 shows d*q*/d*V* curves of the MoO_2.5_/NC and MoO_2.0_N_0.5_/NC as electrodes in a voltage range of 0.1–3 V. During the initial cycle of MoO_2.0_N_0.5_/NC cells, we found three stages of sodiation at ~ 0.7, 1.1 V; while there are four stages of sodiation at ~ 0.6, 0.9, 1.1, 2.5 V for MoO_2.5_/NC cells (observed on Fig. S9a–b). Interestingly, after the initial cycle, the sodiation stage changes to ~ 0.7 V, suggesting that the electrodes undergo irreversible reactions during the initial discharge; however, the reaction observed in further cycles is highly reversible. Importantly, the initial d*q*/d*V* curve of MoO_2.0_N_0.5_/NC exhibits less irreversible reactions, indicating that it is a more stable anode material for sodium storage.

**Fig. 5:**
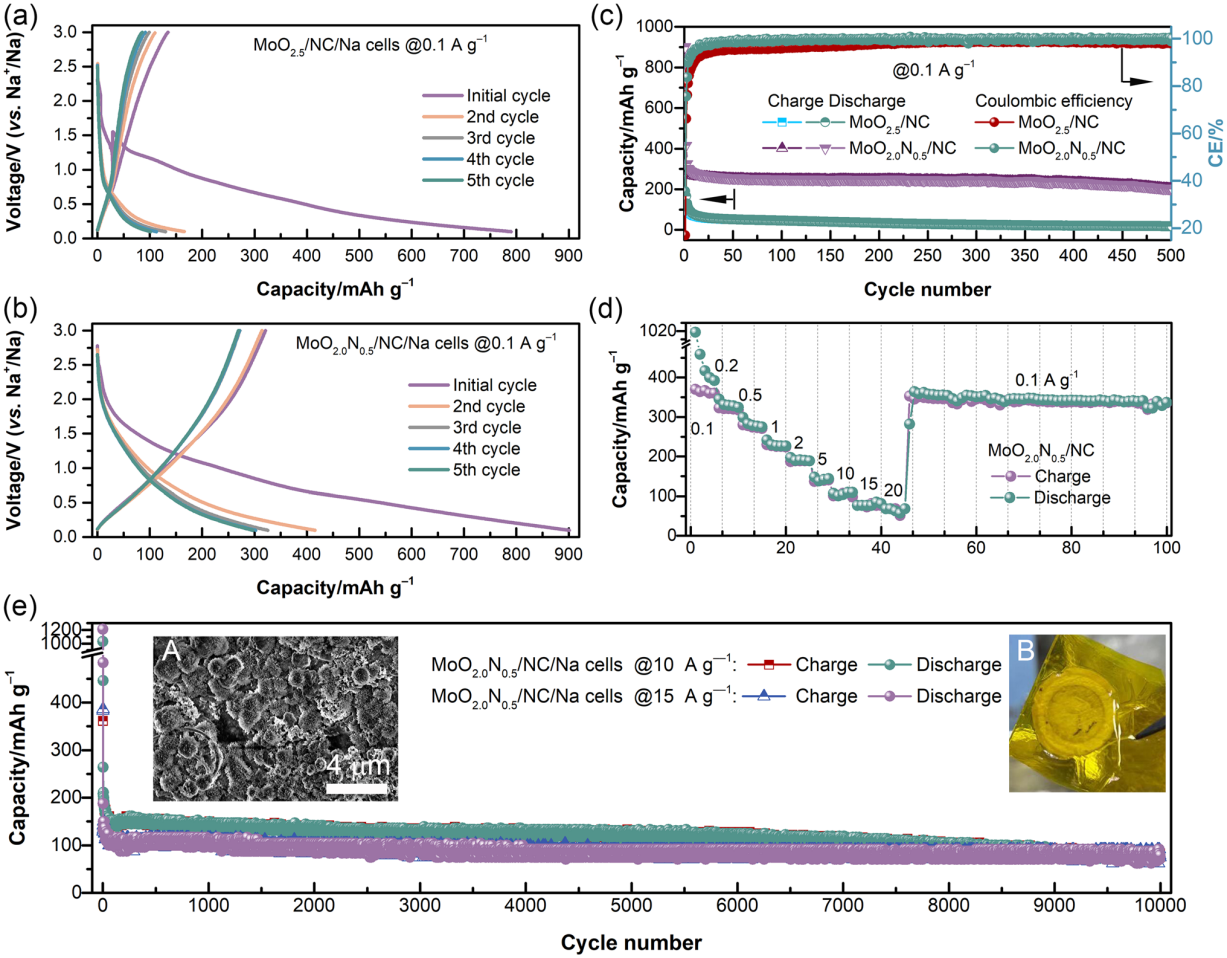
Electrochemical performance of MoO_2.0_N_0.5_/NC electrode. Initial charge and discharge cycles of **a** MoO_2.5_/NC and **b** MoO_2.0_N_0.5_/NC. **c** Cycling performance of MoO_2.5_/NC and MoO_2.0_N_0.5_/NC. **d** Rate cycling performance of MoO_2.0_N_0.5_/NC. **e** Long-term cycling performance at current densities of 10 and 15 A g^−1^. The insert figures of **e** shown **A** the SEM image of MoO_2.0_N_0.5_/NC electrode after 10,000 cycles at 10 A g^−1^ and **B** the digital picture of separator faced MoO_2.0_N_0.5_/NC electrode side. The MoO_2.0_N_0.5_/NC cell was disassembled after 10,000 cycles at 10 A g.^−1^

The successful synthesis of the MoO_2.0_N_0.5_/NC for a superior SIB anode material is evident from the excellent electrochemical behavior (Fig. 5). Figure 5a–b displays the galvanostatic voltage profiles of MoO_2.5_/NC and MoO_2.0_N_0.5_/NC at initial cycles with a current density of 0.1 A g^−1^, thereby suggesting MoO_2.0_N_0.5_/NC has high reversibility. Furthermore, the results demonstrate that the sloping electrochemical curves of MoO_2.0_N_0.5_/NC are analogous to those materials, which exhibit a solid-solution behavior, *i.e.,* single-phase reaction [49]. The initial charge capacity of MoO_2.0_N_0.5_/NC is 321.0 mAh g^−1^, which is higher than that of MoO_2.5_/NC of 134.6 mAh g^−1^. In the initial cycles, the side reaction is dominantly attributed to the decomposition of the electrolyte forming the SEI layer on the electrode surface. Specifically, the activation process can present low initial Coulombic efficiency (CE). Figure 5c exhibits the cycling performance of the MoO_2.0_N_0.5_/NC electrode in the voltage range of 0.1–3 V (*vs*. Na^+^/Na) with a current density of 0.1 A g^−1^. The results indicate highly reversible capacity and distinct characteristics. The cycling performance reveals an ultra-stable cyclability over 500 cycles at 0.1 A g^−1^ and a CE approach 100% except for initial cycles. The cyclability of MoO_2.0_N_0.5_/NC significantly exceeded that of MoO_2.5_/NC anode at 0.1 A g^−1^ due to the introduction of MoO_2.0_N_0.5_ atomic nanoclusters.

To further evaluate the electrochemical properties of the MoO_2.0_N_0.5_/NC electrode, the C-rate performance and long-term cycling performance are conducted by galvanostatic charge/discharge with various currents (shown in Figs. 5d–e and S10). In the last 5 cycles of each current, reversible charge specific capacity of 360.4, 317.6, 270.8, 224.3, 189.0, 101.1, 76.8, 76.8, and 68.5 mAh g^−1^ is observed at 0.1, 0.2, 0.5, 1, 2, 5, 10, 15, and 20 A g^−1^, respectively. It is notable that when the current is reverted to 0.1 A g^−1^ after 45 cycles, the capacity is restored to the early stage. The excellent rate capability characterizations are ascribed to the metallic electron-conductivity of transition metal nitride, the unique ordered mesoporous structure, and the introduction of MoO_2.0_N_0.5_ atomic nanoclusters [50]. More importantly, the MoO_2.0_N_0.5_/NC electrode also indicates exceptional cycling stability even at higher current densities. The rate cyclability of MoO_2.0_N_0.5_/NC significantly exceeded that of MoO_2.5_/NC (Fig. 5d) and carbonaceous [49] anode materials. As shown in Fig. 5e, the as-fabricated electrode exhibits a high capacity of 83.3 mAh g^−1^ and 79.2 mAh g^−1^ after 10,000 cycles at 10 and 15 A g^−1^, respectively, without noticeable capacity fading. After long-term cycles, the sodium-ion cells are disassembled in an Ar-filled glovebox. The coin cell case, sodium metal electrode, and separators of MoO_2.5_/NC cells display a dark-black color due to amounts of side-reactions, which is the main reason that the cells cannot deliver the expected capacity, while these of MoO_2.0_N_0.5_/NC cells have neglected changes, *i.e.,* less side-reaction, leading to significantly battery performance (Fig. S11 and insert of Fig. 5e). The hollow microsphere structure of MoO_2.0_N_0.5_/NC is well maintained after long-term cycling, indicating that the MoO_2.0_N_0.5_/NC as an anode shows superior stability during sodium-ion intercalation/de-intercalation (insert A of Fig. 5e). The results reveal that MoO_2.0_N_0.5_/NC anode shows excellent stability against the electrolytes and results in superior rate capability and long lifespan, which could be a feasible candidate for energy storage fields since it is economically competitive (Table S2). Table S2 lists a comparison of rate capacity of carbonaceous materials for SIBs, which indicates that battery performance of MoO_2.0_N_0.5_/NC is superior to the most Mo-based anode materials.

**Fig. 6:**
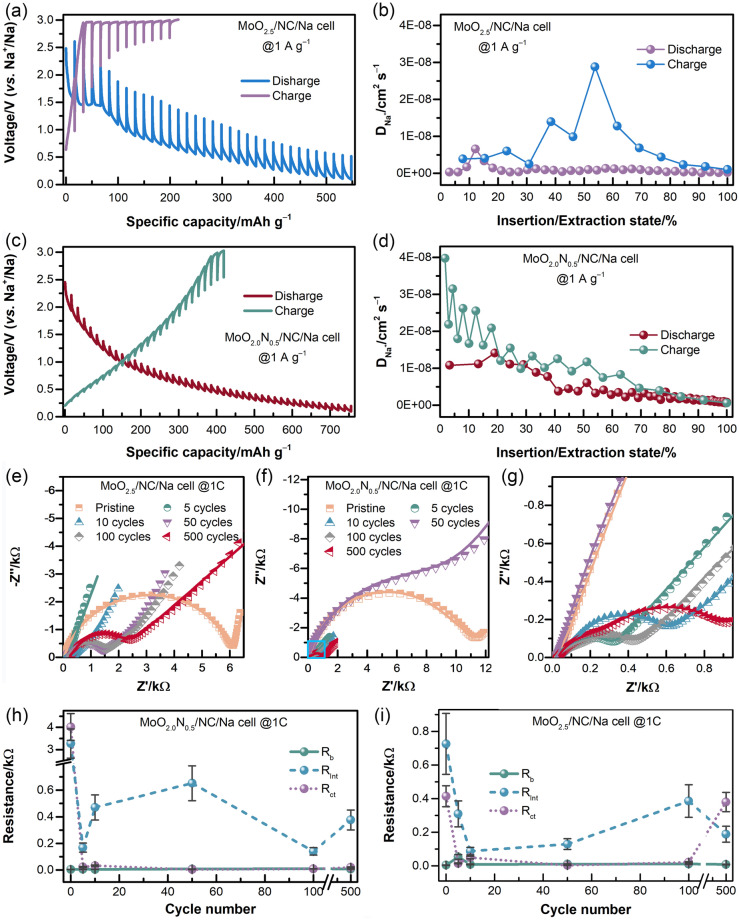
GITT profiles (current pulse at 1 A g^−1^ for charging/discharging with 20 min relaxation) for **a** MoO_2.5_/NC and **c** MoO_2.0_N_0.5_/NC, and corresponding sodium-ion diffusion coefficient of **b** MoO_2.5_/NC and **d** MoO_2.0_N_0.5_/NC electrodes. Nyquist plots of **e** MoO_2.5_/NC, **f–g** MoO_2.0_N_0.5_/NC; the resistance of the cells as functions of cycle number at 1 A g.^−1^: **h** MoO_2.5_/NC, and **i** MoO_2.0_N_0.5_/NC

To analyze the electrochemical kinetics of the MoO_2.5_/NC and MoO_2.0_N_0.5_/NC materials, galvanostatic intermittent titration technique (GITT) and EIS are conducted, and the results display in Fig. 6. The results of GITT test provide that the diffusion coefficient of Na^+^ of MoO_2.5_/NC and MoO_2.0_N_0.5_/NC electrode during charging/discharging is 10^−10^–10^−8^ cm^2^ s^−1^. It can be observed that the diffusion coefficient of sodium ion of MoO_2.0_N_0.5_/NC electrode is considerably much higher than that of MoO_2.5_/NC electrode. The strong dynamic features of MoO_2.0_N_0.5_/NC materials are probably due to the unique structure with MoO_2.0_N_0.5_ atomic nanocluster introducing and N-doping that can promote the Na^+^ migration.

The Nyquist plots are shown in Fig. 6e–g. The Nyquist plots are fitting by an equivalent circuit shown in Fig. S12 and the obtained values of resistance are listed in Table S3. The EIS test results of the fully discharging process demonstrated that the *R*_b_ of the MoO_2.0_N_0.5_/NC/Na cells is maintained at 4.3–8.0 Ω (Table S3), and no evident change can be observed, indicating that the electrochemical reaction process of MoO_2.0_N_0.5_/NC is reversible. In the early stage, the decrease *R*_ct_ is possible due to the influence of SEI film. In the later stage, the *R*_b_ and *R*_ct_ of MoO_2.0_N_0.5_/NC are smaller than that of MoO_2.5_/NC, indicating that MoO_2.0_N_0.5_/NC electrodes have the faster electron transfer rate (*i.e.,* the higher conductivity) and more stable nanosheet structure [51, 52]. The *R*_int_ of MoO_2.5_/NC/Na and MoO_2.0_N_0.5_/NC/Na cells decreases first, and slightly fluctuates around 0.3 Ω, indicating sodium ion between the electrode materials and current collector has no significant difference [53].

Figure S13 shows the Bode plots of MoO_2.0_N_0.5_/NC and MoO_2.5_/NC after various cycles with fully discharged. The Nyquist plots consist of two integrated semicircles at high and medium frequency range and an almost vertical line at low-frequency range (Fig. 6e–g). The semicircles are attributed to interfacial resistance and bulk resistance, while the vertical line at low-frequency range is assigned to sodium-ion diffusion [53]. As shown in Fig. S14a, there is one peak between 1 and 100 kHz, while there are two peaks at this frequency range, which can be explained that MoO_2.0_N_0.5_/NC has higher specific surface area than MoO_2.5_/NC and is line with analysis on N_2_-sorption isotherms, resulting in a higher surface activity and forming a thicker SEI layer at ~ 100 kHz [54]. The main difference between MoO_2.0_N_0.5_/NC and MoO_2.5_/NC is that MoO_2.0_N_0.5_/NC has a peak at ~ 1 kHz, which is contributed to the better capacity of electron migration. Moreover, the angle phase of MoO_2.0_N_0.5_/NC at low-frequency region is slightly smaller than that of MoO_2.5_/NC, illustrating that MoO_2.0_N_0.5_/NC has a faster sodium-ion diffusion in the electrodes [39].

## Conclusion

In summary, we have demonstrated a two-step self-templating strategy for the synthesis of a novel hybrid architecture composed of MoO_2.0_N_0.5_ atomic nanoclusters bonded in nanosheets of N-doped carbon hierarchical hollow microspheres (MoO_2.0_N_0.5_/NC). The synthetic method can readily regulate the composition and structure of hybrid materials. With the inherent advantages of MoO_2.0_N_0.5_ atomic nanoclusters and the unique physicochemical characteristic of the three-dimensional hybrid host, the MoO_2.0_N_0.5_/NC anode empowers with both prolonged lifespan and enhanced rate capability, which satisfies the requirement of anode materials for the commercialization of sodium storage. This work may provide a feasible synthesis solution and structural perspective of transition metal nitrides atomic nanoclusters to be applied for sodium-ion batteries.

## Supplementary Information

Below is the link to the electronic supplementary material.

Supplementary file1 (PDF 1656 kb)
