# Supplementary material for: Molybdenum Oxynitride Atomic Nanoclusters Bonded in Nanosheets of N-Doped Carbon Hierarchical Microspheres for Efficient Sodium Storage
Source: Nanomicro Lett. 2022 Aug 13;14:163. doi: 10.1007/s40820-022-00893-7 (PMC9375813; doi:10.1007/s40820-022-00893-7)
Supplement: Supplementary file 1 — Supplementary file1 (PDF 1656 kb) [file 40820_2022_893_MOESM1_ESM.pdf]

Supporting Information for

Molybdenum Oxynitride Atomic Nanoclusters Bonded in Nanosheets of N-Doped Carbon Hierarchical Microspheres for Efficient Sodium Storage

Xiaona Pan[1], Baojuan Xi[1, *], Huibing Lu[1], Zhengchunyu Zhang[1], Xuguang An[2], Jie Liu[3], Jinkui Feng[4], and Shenglin Xiong[1, *]

[1]School of Chemistry and Chemical Engineering, State Key Laboratory of Crystal Materials, Shandong University, Jinan 250100, P. R. China

[2]School of Mechanical Engineering, Chengdu University, Chengdu 610106, P. R. China

[3]The State Key Laboratory of High Performance Ceramics and Superfine Microstructure, Shanghai Institute of Ceramics, Chinese Academy of Sciences, Shanghai 200050, P. R. China

[4]School of Materials Science and Engineering, Shandong University, Jinan 250100, P. R. China

*Corresponding authors. E-mail: basojuanxi@sdu.edu.cn (Baojuan Xi), chexsl@sdu.edu.cn (Shenglin Xiong)

Supplementary Figures and Tables

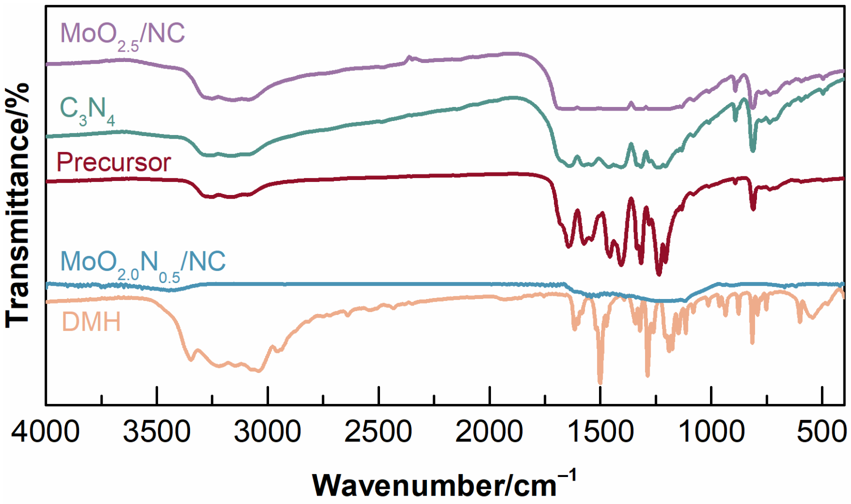

Fig. S1 FT-IR spectra for dopamine hydrochloride (DMH), Mo-polydopamine hybrid (precursor), C_3N_4, $MoO_{2.5}$/NC and $MoO_{2.0}N_{0.5}$/NC powder in a range of 4000-400 cm^{-1}

Note: As shown in Fig. S1, curves are FT-IR spectra of dopamine hydrochloride (DMH), Mo-polydopamine hybrid (precursor), C_3N_4, and prepared $MoO_{2.5}$/NC and $MoO_{2.0}N_{0.5}$/NC powder. The FT-IR spectra of DMH, it can be observed that the adsorption peaks at 1342, 1319, 1188, and 1174 cm^{-1} are assigned to CH_2, C-O-H bending vibration, C-O and C-C stretching vibration, respectively [S1]. As for FT-IR spectra of the precursor, there are new adsorption peaks appearing at 1534 and 1429 cm^{-1}, indicating that the indole structure is formed [S2].

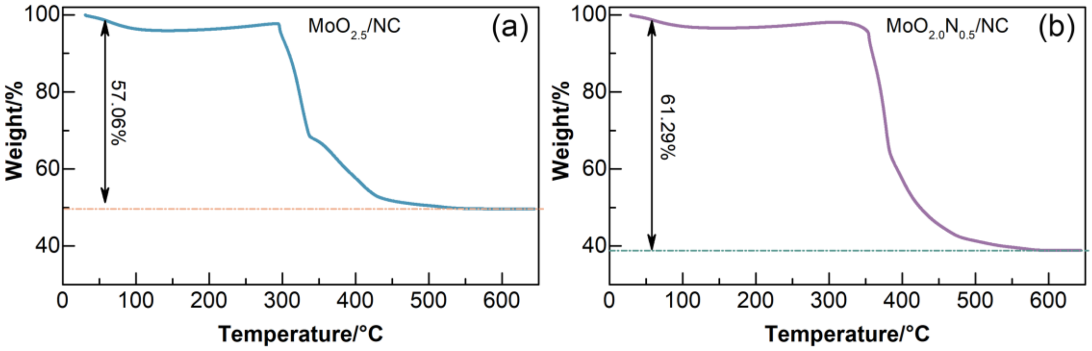

Fig. S2 TGA curves for **a** $MoO_{2.5}$/NC and **b** $MoO_{2.0}N_{0.5}$/NC

Note: The content of carbon in $MoO_{2.5}$/NC and $MoO_{2.0}N_{0.5}$/NC is calculated by TGA test. The calculation equation is shown below:

$$MoO_{2.5}+1/4O_2=MoO_3$$

$$\text{Mass of } MoO_3=143.94a/135.94=1.06a$$

Assuming the mass of $MoO_{2.5}$ is a, we can obtain the mass of MoO_3 should be 1.06a, indicating that complete combustion of the purity $MoO_{2.5}$ in the air would have a 6% weight increase.

$$MoO_{2.5}/NC+5/4O_2=MoO_3+CO_2$$

Here, we assume the content of carbon in $MoO_{2.5}$ is x, the weight loss of $MoO_{2.5}$/NC is 50.49%.

$$x-6\%(1-x)=50.49\%$$

The value of x is obtained, *i.e.* the content of carbon in $MoO_{2.5}$/NC is 53.3%.

By this method, we calculated that the carbon content of $MoO_{2.0}N_{0.5}$/NC is about 62.9%.

Table S1 The overall content of C, H, N, and Mo elements in the $MoO_{2.5}$/NC and $MoO_{2.0}N_{0.5}$/NC powder materials

Name	C^a/%	N^a/%	Mo^b/%	O/%
$MoO_{2.5}$/NC	50.69	0.33	34.82	14.25
$MoO_{2.0}N_{0.5}$/NC	54.37	2.36	32.46	10.81

[a]Obtained from CHN elemental analysis; [b]Estimated by ICP-MS measurement

Note: Table S1 lists the main element content of as-prepared materials. By the calculation, we obtained Mo/O of 1/2.5 and Mo/O/N of 1/2.0/0.5 for the materials, *i.e.* $MoO_{2.5}$/NC and $MoO_{2.0}N_{0.5}$/NC. The results are verified in the following discussion.

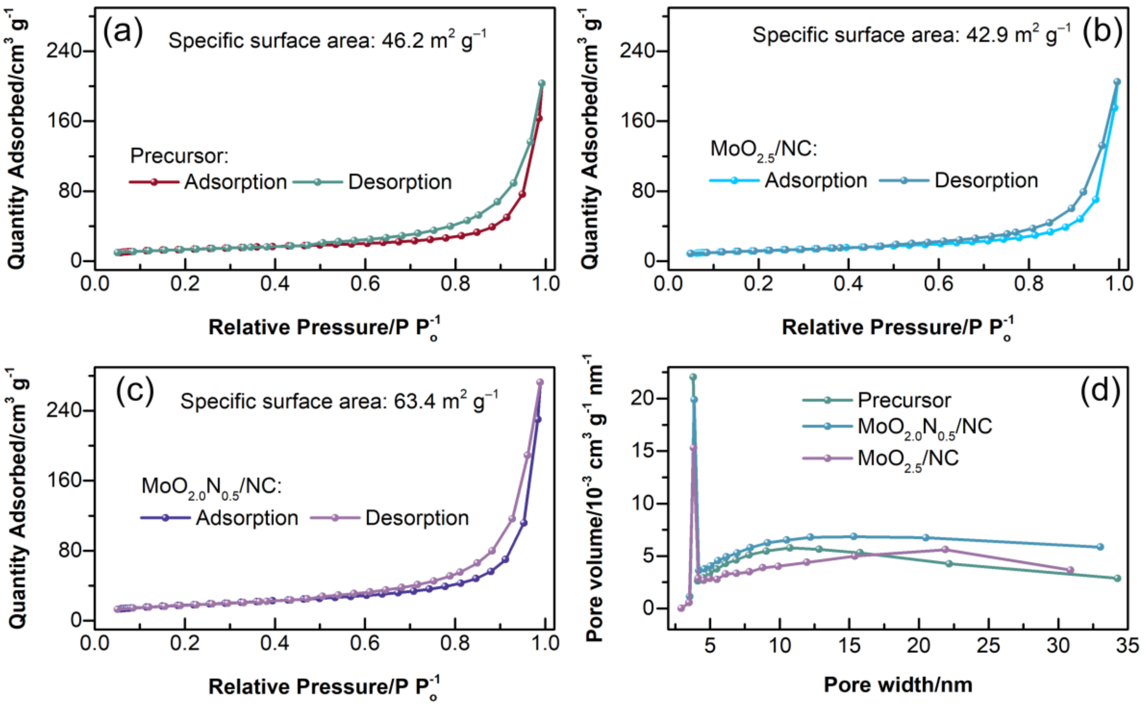

Fig. S3 N_2 sorption isotherms of **a** precursor, **b** $MoO_{2.5}$/NC, **c** $MoO_{2.0}N_{0.5}$/NC, and **d** pore size distribution

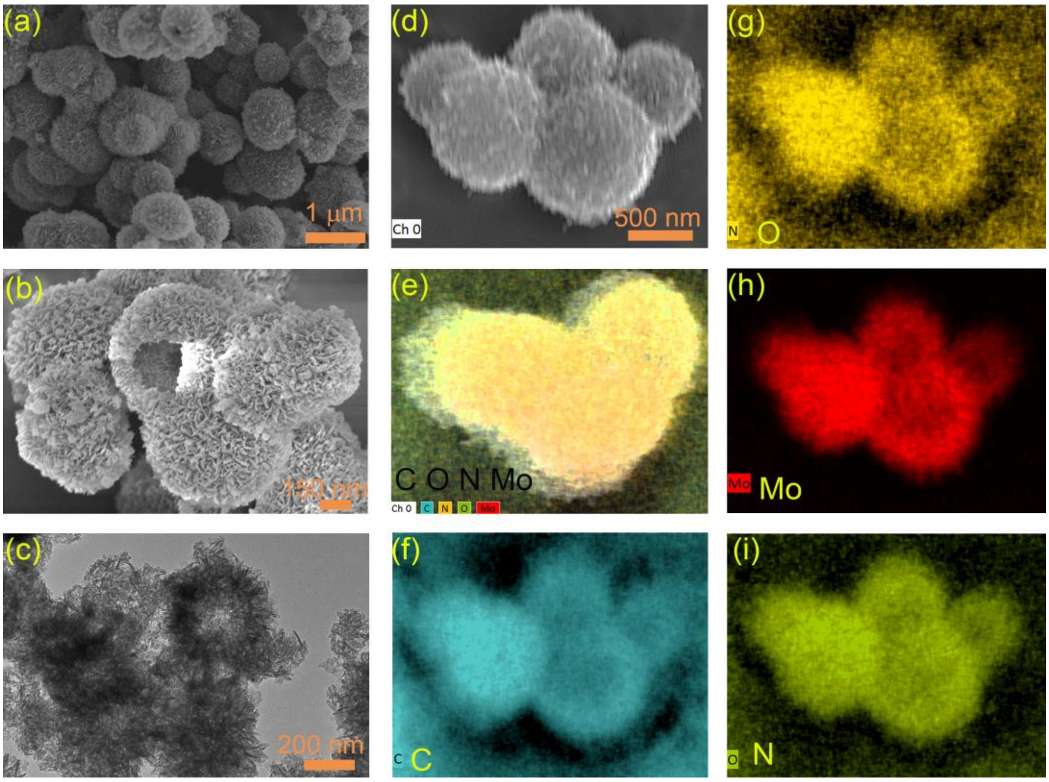

Fig. S4 Morphology and EDS mappings for the precursor of $MoO_{2.5}$/NC and $MoO_{2.0}N_{0.5}$/NC powder. **a**, **b** FESEM images for the precursor of $MoO_{2.5}$/NC and $MoO_{2.0}N_{0.5}$/NC powder; **c** TEM images for the precursor of $MoO_{2.5}$/NC and $MoO_{2.0}N_{0.5}$/NC powder; and **d-i** FESEM images, and the corresponding EDS mappings of **f** C, **g** O, **h** Mo, and **i** N

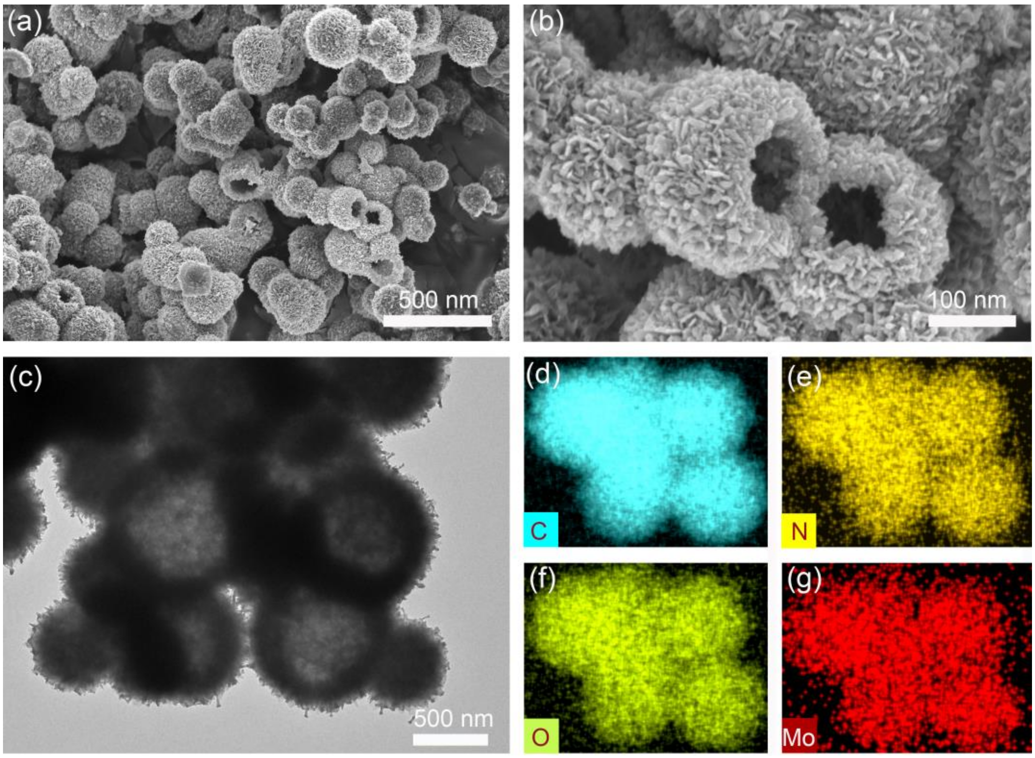

Fig. S5 Morphology and EDX mappings for the precursor of $MoO_{2.5}$/NC powder. **a**, **b** SEM images for the $MoO_{2.5}$/NC powder; **c** TEM images for the $MoO_{2.5}$/NC powder; and **d-g** EDS mappings of **d** C, **e** N, **f** O, and **g** Mo. As the SEM and TEM images shown, it is obvious observed that the $MoO_{2.5}$/NC maintains the hollow microsphere after the precursor calcined at elevated temperature

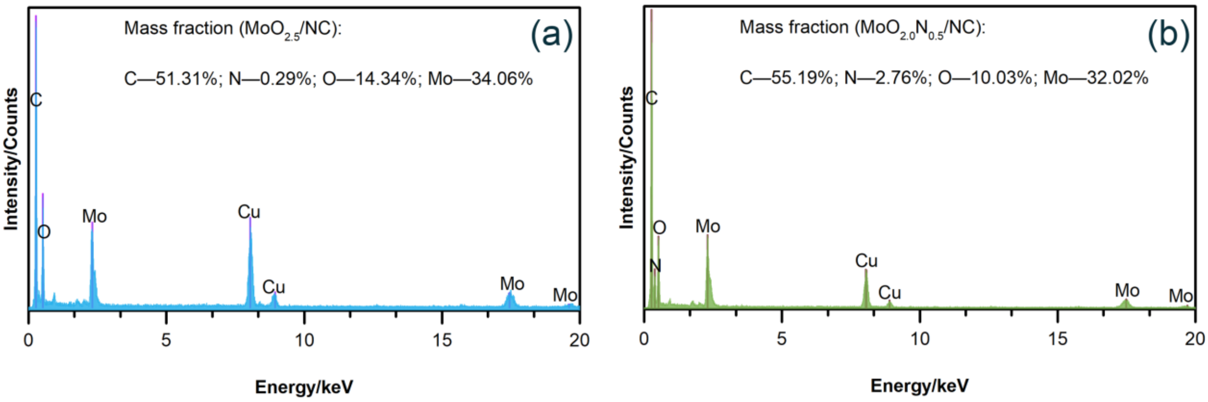

Fig. S6 EDX spectra under TEM of $MoO_{2.5}$/NC **a** and $MoO_{2.0}N_{0.5}$/NC **b** powder materials. The copper signal is from the TEM sample grid

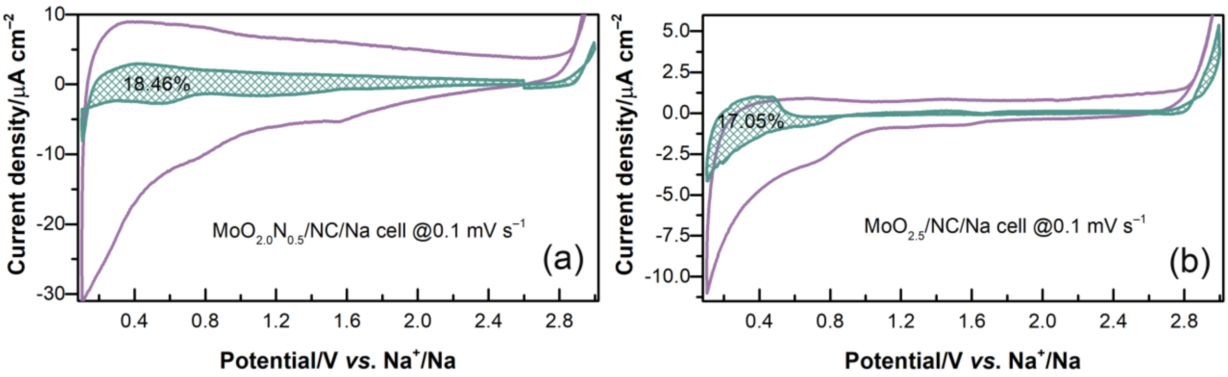

Fig. S7 The capacitive contribution ratio of $MoO_{2.5}$/NC **a** and $MoO_{2.0}N_{0.5}$/NC **b** with a scan rate of 0.1 mV s^{-1}

Note: As shown in Fig. S7, about 17.05% and 18.46% capacitive contribution can be received from *i*(V).

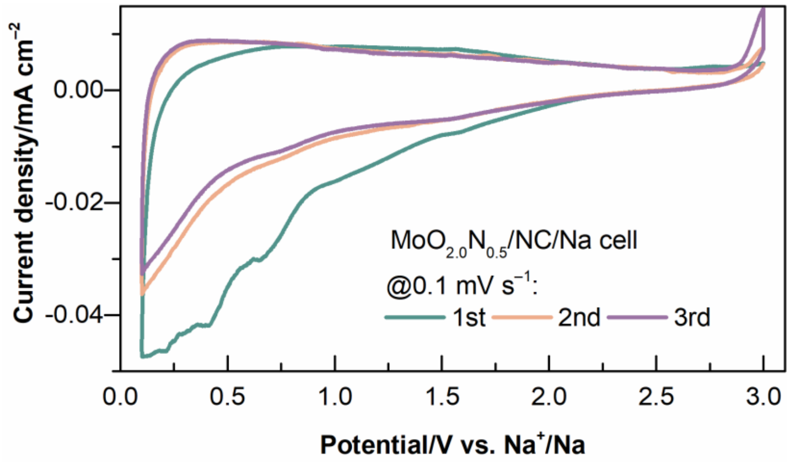

Fig. S8 CV curves for $MoO_{2.0}N_{0.5}$/NC/Na cell with a scan rate of 0.1 mV^{-1}

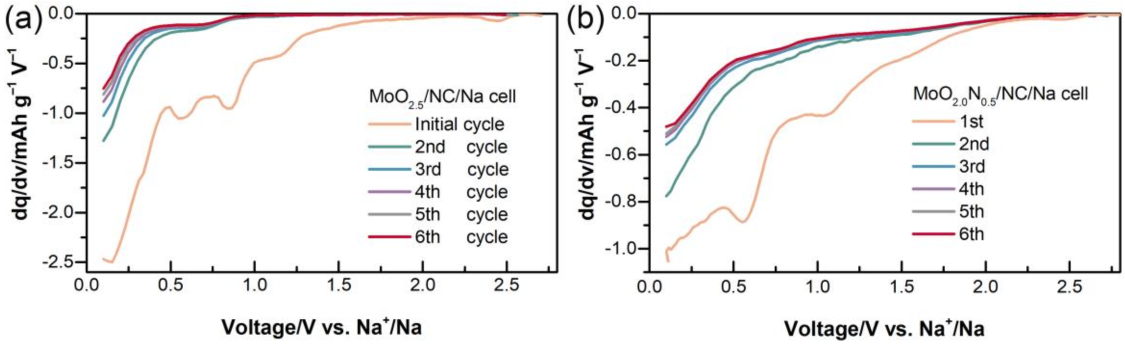

Fig. S9 Differential capacity curves (d*q*/d*V*) corresponding to the voltage profiles in Fig. 5**a** and **b** with expanded scales to emphasize the electrochemical features: **a** $MoO_{2.5}$/NC/Na cell and **b** $MoO_{2.0}N_{0.5}$/NC/Na cell

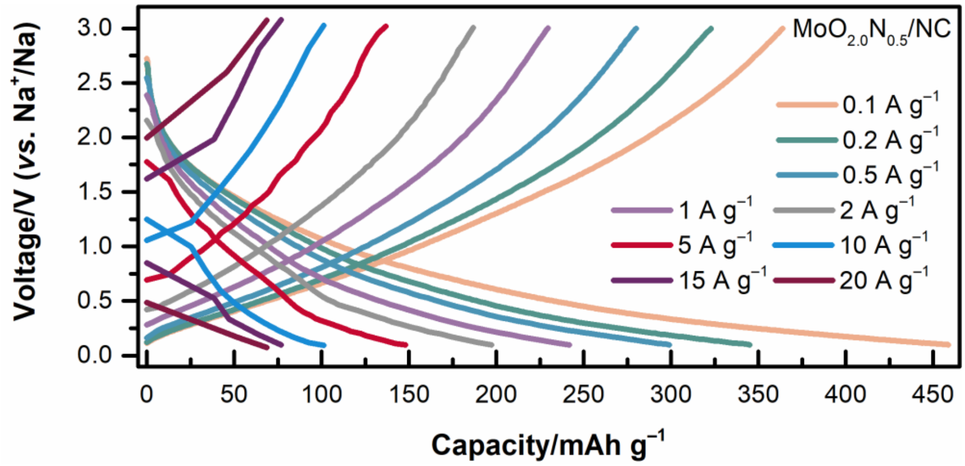

Fig. S10 Galvanostatic charge/discharge curves of the $MoO_{2.0}N_{0.5}$/NC anode at different current densities

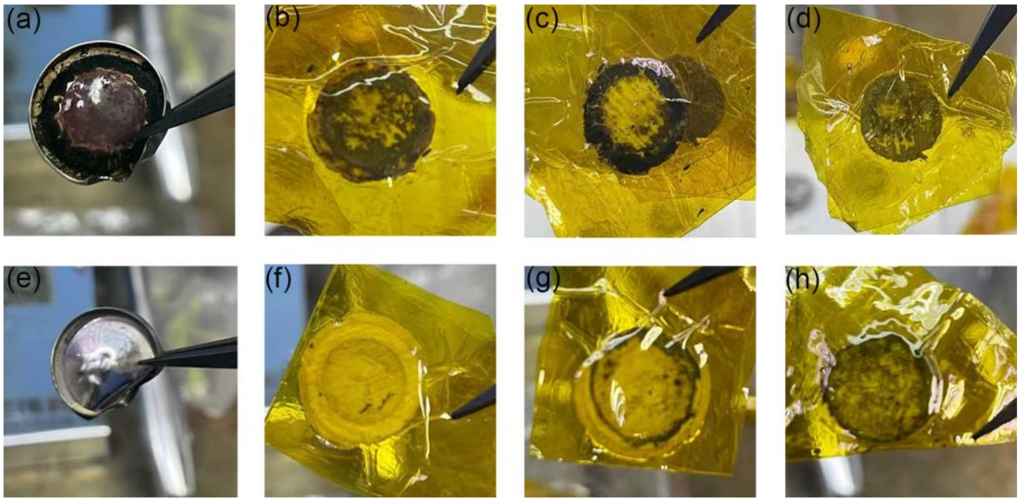

Fig. S11 The digital picture of disassembled sodium-ion cells with **a-d** $MoO_{2.5}$/NC and **e-f** $MoO_{2.0}N_{0.5}$/NC as anodes. The SIBs are cycled for 10,000 cycles with a current density of 1 A g^{-1} in a voltage range of 0.1−3 V (*vs.* Na^+/Na). The coin cell cases **a**, separator faced $MoO_{2.5}$/NC anode side **b**, separator faced sodium metal electrode side **c**, and sodium metal electrode **d** of $MoO_{2.5}$/NC/Na cells exhibit obviously changes. There are amount of side products with dark black color, which results in unexpected specific capacity. In contrast, **e** coin cell cases, **f** separator faced $MoO_{2.0}N_{0.5}$/NC anode side, **g** separator faced sodium metal cathode side, and **h** sodium metal cathode have neglected changes, which leads to excellent cycling performance and rate capability

Table S2 The sodium-ion battery performance of transition metal nitride/oxide electrode. Regularly, the charge/discharge voltage range is 0.01-3 V (*vs.* Na^+/Na). In this work, the voltage range is 0.1-3 V (*vs.* Na^+/Na). a. the specific charge capacity of second cycles

Electrode appellation	Specific Capacity (mAh g^{-1})	Reversible capacity (mAh g^{-1})	Refs.
VN@rGO	400 @50 mA g^{-1}	155 after 10000 cycles @1 A g^{-1}	[S3]
G-VNQD-500	203 @0.2C	230 after 800 cycles @1C	[S4]
Ga_2N	343 @0.05 A g^{-1}	320 after 30 cycles @0.05 A g^{-1}	[S5]
Mo_2N@G-rGO	487.2 @0.2 A g^{-1}	250 after 150 cycles @0.1 A g^{-1}	[S6]

Por-Mo_2N-NBs	214 @0.05 A g^{-1} 79 @2 A g^{-1}	155 after 200 cycles @0.1 A g^{-1}	[S7]
Com-Mo_2N-NPs	134 @0.05 A g^{-1}	~50 after 200 cycles @0.1 A g^{-1}	[S7]
Fe_3N@C yolk-shell	248 @2 A g^{-1}	~285 after 50 cycles @0.05 A g^{-1}	[S8]
Fe_3N@C bulk particles	89 @2 A g^{-1}		[S8]
Meso-Mo_2N	156 @1 A g^{-1}	136 after 1000 cycles @1 A g^{-1}	[S9]
MoO_2@C nanoflower	172 @0.1 A g^{-1} 67 @8 A g^{-1}	166 after 1000 cycles @1 A g^{-1}	[S10]
$MoO_{2.5}$@NC	238 @0.1 A g^{-1} 67 @10 A g^{-1}	115 after 5000 cycles @5 A g^{-1}	[S11]
Mo_2N@C-rGO	490 @0.2 A g^{-1} ~220 @1 A g^{-1}	487 after 150 cycles @0.2 A g^{-1}	[S12]
MoP/C NWs	293 @0.1 A g^{-1} 71 @ 71 A g^{-1}	106 after 3500 cycles @1 A g^{-1}	[S13]
TiN@N-TiO_2	226.9 @0.1 C	198.7 after 500 cycles @0.335 A g^{-1}	[S14]
Meso-Mo_3N_2-NWs	282 @0.1 A g^{-1} 87 @16 A g^{-1}	221.7 after 800 cycles @1 A g^{-1}	[S15]
Mo_2N@NCN	~230 @0.5 A g^{-1}	~180 after 300 cycles @0.5 A g^{-1}	[S16]
MnN@RGO		716 after 180 cycles @0.1 A g^{-1}	[S17]
Cu_3N	277 @0.39 A g^{-1}	70 after 50 cycles @0.39 A g^{-1}	[S18]
ACGC900	250 @0.05 A g^{-1}	210 after 200 cycles @0.05 A g^{-1}	[S19]
GC	180 @0.05 A g^{-1}	150 after 200 cycles @0.05 A g^{-1}	[S19]
AC	150 @0.05 A g^{-1}	50 after 200 cycles @0.05 A g^{-1}	[S19]
mFePA-HS	410 @0.2 A g^{-1}	200 after 50 cycles @0.2 A g^{-1}	[S20]
Blank-FePA	250 @0.2 A g^{-1}	100 after 50 cycles @0.2 A g^{-1}	[S20]
SMGA	190 @0.1 A g^{-1}	150 after 500 cycles @0.1 A g^{-1}	[S21]
S-doped honeycomb C	229 @ @5 A g^{-1}; 690.9 @0.1 A g^{-1}	108 after 500 cycles @5 A g^{-1}	[S22]
SiNP@graphene cage/GNS	2980 @0.1 A g^{-1}	2000 after 60 cycles @0.1 A g^{-1}	[S23]
SiNP/GNS	2500 @0.1 A g^{-1}	1000 after 60 cycles @0.1 A g^{-1}	[S23]
SiNPs	2250 @0.1 A g^{-1}	500 after 60 cycles @0.1 A g^{-1}	[S23]
Na(diglyme)$_2C_{20}$	110 @0.0372 A g^{-1}	100 after 1000 cycles @0.0372 A g^{-1}	[S24]
natural graphite	125 @0.5 A g^{-1}	100 after 2500 cycles @0.5 A g^{-1}	[S25]
graphite	115 @0.2 A g^{-1}	100 after 6000 cycles @0.2 A g^{-1}	[S26]
$MoO_{2.5}$/NC	165.8[a] @0.1 A g^{-1} 59.3[a] @0.5 A g^{-1}	3.9 after 10 000 cycles @0.5 A g^{-1}	**This work**
$MoO_{2.0}N_{0.5}$/NC	415.5[a] @0.1 A g^{-1} 208.3[a] @10 A g^{-1}	83.3 after 10 000 cycles @10 A g^{-1}	**This work**

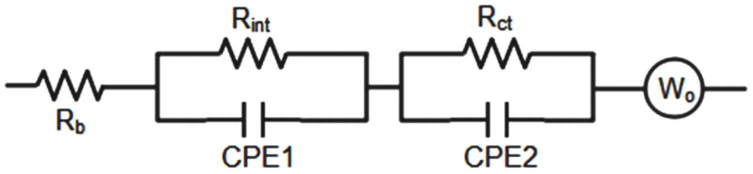

Fig. S12 The equivalent circuit used to fit the EIS Nyquist plots, where the R_b is the bulk resistance of the cells, R_{int} is the interfacial resistance from the two electrolytes/electrode, R_{ct} is the charge transfer resistance

Note: The fitted equivalent circuit is (R_b(R_{int}-CPE1)(R_{ct}-CPE2)W_0) model. The bulk resistance (R_b) of the sodium-ion batteries is assigned to the high frequency area, corresponding to sodium-ion diffusion in the electrolytes. CPE1 is used to replace the double layer between the electrode and the electrolytes due to the roughness of the electrode surface. The interfacial resistance (R_{int}), which is between the electrode and electrolytes, corresponded to semi-cycle of the intermediate frequency. The area of the semi-circle intermediate frequency is mainly composed of charge transfer resistance (R_{ct}) between the GPEs and the active materials layer. CPE2 is generally assigned to the double layer capacitance. A straight line in the low-frequency area is assigned to Warburg impedance (W_0), which corresponded with semi-infinite diffusion of sodium-ion in electrode.

Table S3 The fitting results of electrochemical impedance of $MoO_{2.5}$/NC/Na cells and $MoO_{2.0}N_{0.5}$/NC/Na cells

Electrode materials	Cycle No.	R_b (Ω)	R_{Int} (Ω)	R_{ct} (Ω)
$MoO_{2.5}$/NC	0	4.5	726	414.6
	5	53.5	309.3	14.9
	10	9.0	88.0	50.2
	50	10.3	129.8	3.4
	100	11.3	385.9	21.8
	500	8.7	188.8	380
$MoO_{2.0}N_{0.5}$/NC	0	4.3	3273.0	4017.0
	5	6.6	168.9	23.0
	10	5.2	471.0	33.0
	50	8.2	652.1	4.3
	100	9.5	140.3	9.3
	500	8.0	376.3	20.5

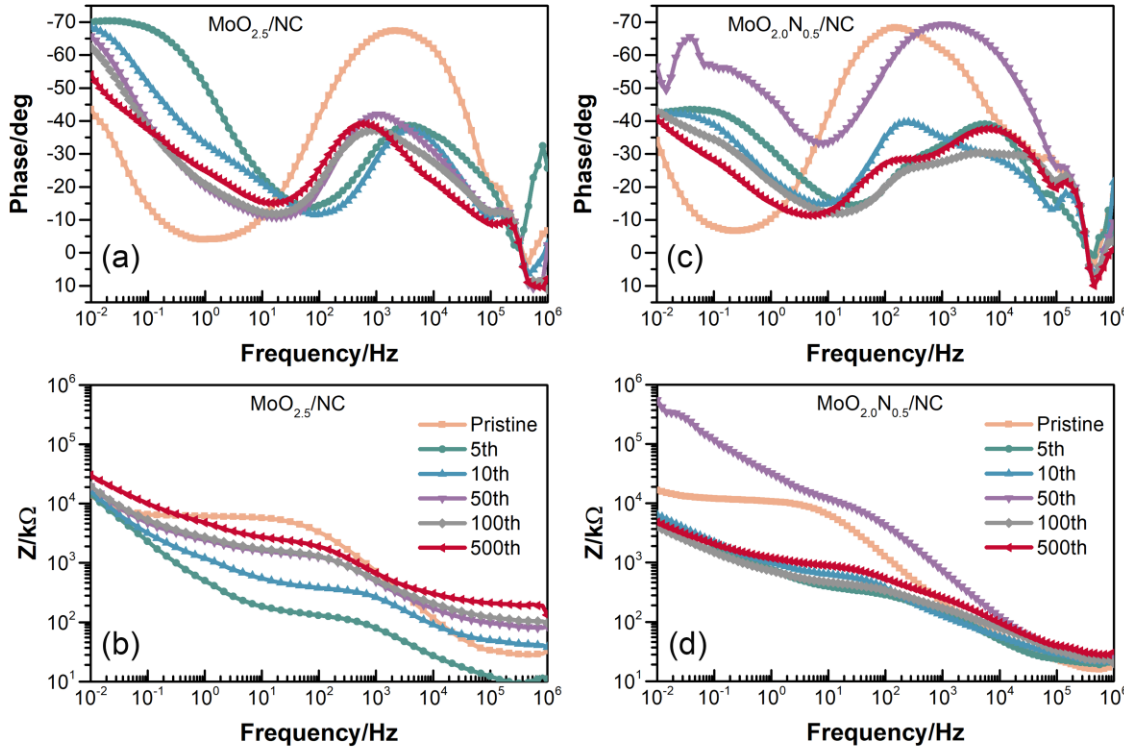

Fig. S13 Bode plots for $MoO_{2.5}$/NC anode **a, b** and $MoO_{2.0}N_{0.5}$/NC anode **c, d** in a range of 0.01 Hz-100 kHz after various fully discharged

Supplementary References

[S1] N. Shi, B. Xi, M. Huang, F. Tian, W. Chen et al., One-step construction of $MoS_{0.74}Se_{1.26}$/N-doped carbon flower-like hierarchical microspheres with enhanced sodium storage. ACS Appl. Mater. Interfaces **11**(47), 44342-44351 (2019). https://doi.org/10.1021/acsami.9b15769

[S2] C. Zhao, J. Kong, L. Yang, X. Yao, S.L. Phua et al., The dopamine–Mo^{VI} complexation-assisted large-scale aqueous synthesis of a single-layer MoS_2/carbon sandwich structure for ultrafast, long-life lithium-ion batteries. Chem. Commun. **50**(68), 9672-9675 (2014). https://doi.org/10.1039/C4CC04099F

[S3] S. Wei, C. Wang, S. Chen, P. Zhang, K. Zhu et al., Dial the mechanism switch of VN from conversion to intercalation toward long cycling sodium-ion battery. Adv. Energy Mater. **10**(12), 1903712 (2020). https://doi.org/10.1002/aenm.201903712

[S4] L. Wang, J. Sun, R. Song, S. Yang, H. Song, Hybrid 2D–0D graphene–VN quantum dots for superior lithium and sodium storage. Adv. Energy Mater. **6**(6), 1502067 (2016). https://doi.org/10.1002/aenm.201502067

[S5] G. Chen, Y. Bai, H. Li, Y. Li, Z. Wang et al., Multilayered electride Ca_2N electrode via compression molding fabrication for sodium ion batteries. ACS Appl. Mater. Interfaces **9**(8), 6666-6669 (2017). https://doi.org/10.1021/acsami.6b16186

[S6] S. Vadahanambi, H. Park, Carbon sheathed molybdenum nitride nanoparticles anchored on reduced graphene oxide as high-capacity sodium-ion battery anodes and supercapacitors. New J. Chem. **42**(8), 5668-5673 (2018). https://doi.org/10.1039/C7NJ04764A

[S7] S. Liu, J. Huang, J. Liu, M. Lei, J. Min et al., Porous Mo_2N nanobelts as a new anode material for sodium-ion batteries. Mater. Lett. **172**(1), 56-59 (2016). https://doi.org/10.1016/j.matlet.2016.02.121

[S8] Z. Li, Y. Fang, J. Zhang, X. Wen, D. Lou, Necklace-like structures composed of Fe_3N@C yolk–shell particles as an advanced anode for sodium-ion batteries. Adv. Mater. **30**(30), 1800525 (2018). https://doi.org/10.1002/adma.201800525

[S9] F. Xu, H. Wang, Mesoporous thin-wall molybdenum nitride for fast and stable Na/Li storage. ACS Appl. Energy Mater. **11**(44), 41188-41195 (2019). https://doi.org/10.1021/acsami.9b07060

[S10] C. Cui, Q. Wei, L. Zhou, L. Mai, J. Ma, Facile synthesis of MoO_2@C nanoflowers as anode materials for sodium-ion batteries. Mater. Res. Bull. **94**(10), 122-126 (2017). https://doi.org/10.1016/j.materresbull.2017.05.046

[S11] B. Huang, S. Liu, X. Zhao, Y. Li, J. Yang et al., Enhancing sodium-ion storage performance of MoO_2/N-doped carbon through interfacial Mo-N-C bond. Sci. China Mater. **64**(1), 85-95 (2021). https://doi.org/10.1007/s40843-020-1370-x

[S12] J. Yuan, X. Hu, J. Chen, Y. Liu, T. Huang et al., In situ formation of vanadium nitride quantum dots on N-doped carbon hollow spheres for superior lithium and sodium storage. J. Mater. Chem. A **7**(15), 9289-9296 (2019). https://doi.org/10.1039/C8TA12512K

[S13] R. Fei, H. Wang, Q. Wang, R. Qiu, S. Tang et al., In situ hard-template synthesis of hollow bowl-like carbon: a potential versatile platform for sodium and zinc ion capacitors. Adv. Energy Mater. **10**(47), 2002741 (2020). https://doi.org/10.1002/aenm.202002741

[S14] X. Ma, J. Tian, F. Zhao, J. Yang, B. Wang, Conductive TiN thin layer-coated nitrogen-doped anatase TiO_2 as high-performance anode materials for sodium-ion batteries. Ionics **24**(4), 3771-3779 (2018). https://doi.org/10.1007/s11581-018-2542-2

[S15] Y. Jiang, J. Dong, S. Tan, Q. Wei, F. Xiong et al., Surface pseudocapacitance of mesoporous Mo_3N_2 nanowire anode toward reversible high-rate sodium-ion storage. J. Energy Chem. **55**(4), 295-303 (2021). https://doi.org/10.1016/j.jechem.2020.07.011

[S16] Y. Li, Y. Yang, J. Zhou, S. Lin, Z. Xu et al., Coupled and decoupled hierarchical carbon nanomaterials toward high-energy-density quasi-solid-state Na-Ion hybrid energy storage devices. Energy Storage Mater. **23**(12), 530-538 (2019). https://doi.org/10.1016/j.ensm.2019.04.007

[S17] V. Sridhar, H. Park, Manganese nitride stabilized on reduced graphene oxide substrate for high performance sodium ion batteries, super-capacitors and EMI shielding. J. Alloys Compd. **808**(5), 151748 (2019). https://doi.org/10.1016/j.jallcom.2019.151748

[S18] X. Li, A.L. Hector, J.R. Owen, Evaluation of Cu_3N and CuO as negative electrode materials for sodium batteries. J. Phys. Chem. C **118**(51), 29568-29573 (2014). https://doi.org/10.1021/jp509385w

[S19] J. Yang, X. Wang, W. Dai, X. Lian, X. Cui et al., From micropores to ultra-micropores inside hard carbon: toward enhanced capacity in room-/low-temperature sodium-ion storage. Nano-Micro Lett. **13**, 98 (2021). https://doi.org/10.1007/s40820-020-00587-y

[S20] Y. Ai, Y. You, F. Wei, X. Jiang, Z. Han et al., Hollow bio-derived polymer nanospheres with ordered mesopores for sodium-ion battery. Nano-Micro Lett. **12**, 31 (2020). https://doi.org/10.1007/s40820-020-0370-1

[S21] F. Song, J. Hu, G. Li, J. Wang, S. Chen et al., Room-temperature assembled MXene-based aerogels for high mass-loading sodium-ion storage. Nano-Micro Lett. **14**, 37 (2022). https://doi.org/10.1007/s40820-021-00781-6

[S22] H. Wan, X. Hu, Sulfur-doped honeycomb-like carbon with outstanding electrochemical performance as an anode material for lithium and sodium ion batteries. J. Colloid Interface Sci. **558**, 242-250 (2019). https://doi.org/10.1016/j.jcis.2019.09.124

[S23] X.Y. Han, D.L. Zhao, W.J. Meng, H.X. Yang, M. Zhao et al., Graphene caging silicon nanoparticles anchored on graphene sheets for high performance Li-ion batteries. Appl. Surf. Sci. **484**, 11-20 (2019). https://doi.org/10.1016/j.apsusc.2019.04.100

[S24] B. Jache, P. Adelhelm, Use of graphite as a highly reversible electrode with superior cycle life for sodium-ion batteries by making use of co-intercalation phenomena. Angew. Chem. Int. Ed. **53**(38), 10169-10173 (2014). https://doi.org/10.1002/anie.201403734

[S25] H. Kim, J. Hong, Y. Park, J. Kim, I. Hwang, Sodium storage behavior in natural graphite using ether-based electrolyte systems. Adv. Funct. Mater. **25**(4), 534-541 (2014). https://doi.org/10.1002/adfm.201402984

[S26] Z. Zhu, F. Cheng, Z. Hu, Z. Niu, J. Chen, Highly stable and ultrafast electrode reaction of graphite for sodium ion batteries. J. Power Sources **293**, 626-634 (2015). https://doi.org/10.1021/jp509385w
